# *In Silico* Molecular Characterization of Human TMPRSS2 Protease Polymorphic Variants and Associated SARS-CoV-2 Susceptibility

**DOI:** 10.3390/life12020231

**Published:** 2022-02-03

**Authors:** Mohd Zulkifli Salleh, Zakuan Zainy Deris

**Affiliations:** Department of Medical Microbiology & Parasitology, School of Medical Sciences, Universiti Sains Malaysia Health Campus, Kubang Kerian 16150, Malaysia; m.z.salleh@usm.my

**Keywords:** SARS-CoV-2, COVID-19, TMPRSS2, spike protein, polymorphisms, susceptibility, molecular docking, serine protease inhibitors, camostat mesylate, nafamostat

## Abstract

The 2019 coronavirus disease (COVID-19) pandemic continues to challenge health care systems worldwide. Severe acute respiratory syndrome coronavirus 2 (SARS-CoV-2) has been responsible for the cause of global pandemic. Type 2 transmembrane serine protease (TMPRSS2) is important in the cell entry and spread of SARS-CoV-2 and plays a crucial role in the proteolytic cleavage of SARS-CoV-2 spike (S) glycoprotein. Here, using reported structural data, we analyzed the molecular complex of TMPRSS2 and the S glycoprotein and further examined intermolecular interactions of natural TMPRSS2 polymorphic variants. We identified several TMPRSS2 variants that could possibly alter host susceptibility to the SARS-CoV-2 infection. Molecular docking analysis revealed that G462D/G462S variants were predicted to be protective variants, whereas Q438E and S339F variants were predicted to increase susceptibility. In addition, we examined intermolecular interactions between TMPRSS2 and its two potential serine protease inhibitors, camostat mesylate and nafamostat. Further, we investigated the effect of TMPRSS2 variants on these interactions. Our structural analysis revealed that G462D, C297S and S460R variants had possibly altered the interactions with the protease inhibitors. Our results identified important TMPRSS2 variations that could be useful to develop high affinity and personalized drugs for treating COVID-19 patients.

## 1. Introduction

The current global coronavirus disease 2019 (COVID-19) pandemic, which is being caused by severe acute respiratory syndrome coronavirus 2 (SARS-CoV-2), has resulted in significant health burden and infected more than 331 million people, with more than 5.5 million deaths worldwide (as of January 2022, WHO). The beta-coronavirus SARS-CoV-2 belongs to the subfamily *Coronavirinae* of the family *Coronaviridae* and the order *Nidovirales*. Likewise, severe acute respiratory syndrome coronavirus (SARS-CoV) and Middle East respiratory syndrome coronavirus (MERS-CoV) are the other two pathogenic coronaviruses that have caused major deadly pneumonic pandemics in the 21st century [1,2]. Like other coronaviruses, SARS-CoV-2 is a positive-strand single-stranded RNA virus, with a linear piece of RNA of approximately 30,000 bases [3]. SARS-CoV-2 virion is composed of four structural proteins, namely the spike (S), envelope (E), membrane (M) and nucleocapsid (N) proteins, in which the N protein wraps around the RNA genome, encapsulated within an envelope that is associated with the S, E and M proteins [4,5]. The transmembrane S glycoprotein of SARS-CoV-2 consists of two subunits, namely the S1 and S2 subunits, and facilitates coronavirus entry into host cells by binding to its receptor, angiotensin-converting enzyme 2 (ACE2), through the receptor-binding domain (RBD) of the S1 subunit [6,7]. The binding activates proteolytic cleavage by type 2 transmembrane serine protease (TMPRSS2) near the junction between the S1 and S2 subunits and subsequently leads to host membrane fusion through irreversible conformational changes, mediated by its S2 subunit [8].

SARS-CoV-2, like any other viruses, accumulates mutations over time, especially in the S glycoprotein. While most of the mutations have little impacts on viral fitness, some confer selective advantages, such as increased transmissibility and infectivity as well as decreased host immune response. Mutations induce alterations in the protein structures of the S glycoprotein, changing the antigenic properties of the strain, which can contribute to reduced effectiveness of an immune response against the original strain [9,10]. SARS-CoV-2 Alpha (B.1.1.7), Beta (B.1.351), Gamma (P.1) and Delta (B.1.617.2) variants are designated as the main variants of concern (VOC) due to their abilities to potentially decrease neutralization by neutralizing antibodies (nAbs) and vaccinations as well as their increased transmissibility, associated with their multiple mutations in the S glycoprotein [11,12,13,14]. The emergence of the new Omicron (B.1.1.529) variant, which was designated as a VOC on 26 November 2021, is of particular concern because the variant harbors a large number of mutations, particularly 30 mutations in the S glycoprotein—half of which are located in the RBD [15]. Some concerning S mutations are K417N, T478K, N501Y and D614G, all of which are found in the previous VOCs and responsible in the increased viral transmission and enhanced immune invasion [9,16]. Moreover, H655Y and N679K mutations are located near the furin cleavage site and may increase S cleavage [17]. This is concerning as these variants may render currently deployed vaccines less effective, thus achieving herd immunity by vaccinations would therefore be more challenging.

The SARS-CoV-2 S glycoprotein and its receptor ACE2 as well as the protease TMPRSS2 play major roles in the viral infection and immune evasion. Mutations and genetic differences in the S glycoprotein, ACE2 and TMPRSS2, all of which responsible for cellular viral entry which might alter the observed infection responses among different individuals. There are several reports show that genetic differences could contribute to different susceptibility to the virus, which cause certain populations—especially those with underlying risk factors—to be more likely to suffer from the viral infection [18,19,20,21]. More importantly, gender-specific differences between males and females that exhibit sexual dimorphism also contribute to different susceptibility to the infection, disease progression and treatment [22]. While both sexes have the same COVID-19 prevalence, men with the disease are more likely to be high risk, leading to severe prognosis and death, regardless of age [23,24]. High concentrations of testosterone enhance TMPRSS2 levels which may lead to higher disease susceptibility in males [25]. Furthermore, like gender, age also plays an important role in the disease’s progression. Children and young adults with COVID-19 are generally asymptomatic and less severe in contrast to older people [26]. Different clinical characteristics of COVID-19 in children and young adults compared to mature adults may be due to the different expressions of genetic components, such as ACE2 and TMPRSS2 [27,28,29].

An analysis of the human ACE2 polymorphisms on its binding to the S glycoprotein was recently published. The study provides valuable insights into natural ACE2 variations that have increased or decreased its affinity to the RBD of the S glycoprotein, which may confer protection or render individuals more predisposed to the viral infection [30]. Interactions between the RBD and its ACE2 receptor are crucial for the recognition and cellular entry of the virus [7]. Similarly, TMPRSS2 plays a major role in the SARS-CoV-2 infection and is important for the S glycoprotein priming [8]. Albeit important, structural details of intermolecular interactions between TMPRSS2 and the S glycoprotein are scant.

In this study, using the recently reported structural data of TMPRSS2 [31], we analyzed the molecular complex of TMPRSS2 and the S glycoprotein and expounded structurally in detail the intermolecular interactions of natural TMPRSS2 polymorphic variants. In addition, we investigated the intermolecular interactions between TMPRSS2 and its two potential inhibitors, namely camostat mesylate and nafamostat [32]. These findings provide valuable information on molecular interactions between TMPRSS2 and the S glycoprotein. Understanding these interactions at the molecular level will be helpful to the development of therapeutics against SARS-CoV-2, which selectively inhibit TMPRSS2 and other serine proteases, in order to fight the COVID-19 threat.

## 2. Methodology

### 2.1. Structure Retrieval, Molecular Modelling and Docking

**Figure 1:**
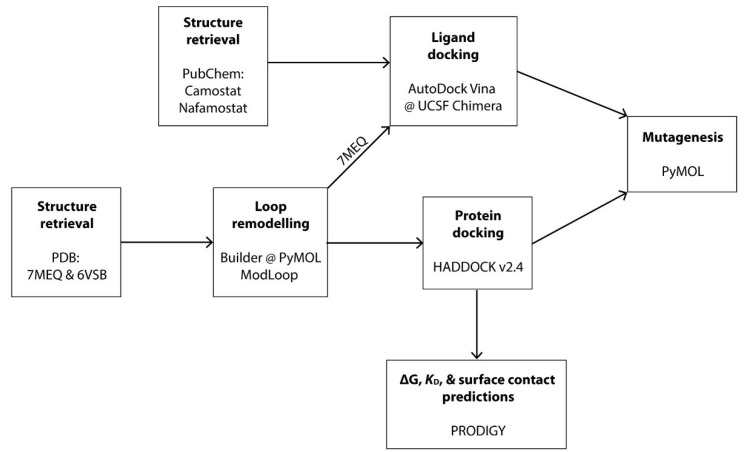
Schematic diagram of methodology used in this study.

The atomic coordinates of the human TMPRSS2 and SARS-CoV-2 S glycoprotein used in this study were retrieved from the RCSB Protein Data Bank (PDB) and the accession numbers were 7MEQ [31] and 6VSB [33], respectively. Both structures were subjected to manual inspection; ligands were removed, and all gaps and missing loops were built manually using Builder, as implemented in PyMOL. Loops subsequently were refined and modelled using the automated loop modelling web server, ModLoop [34], available at https://modbase.compbio.ucsf.edu/modloop/ (accessed on 28 November 2021). Both the TMPRSS2 and S protein structures were subjected to molecular docking using the HADDOCK 2.4 web server [35], available at https://wenmr.science.uu.nl/haddock2.4/ (accessed on 30 November 2021). Two docking simulations were run for each cleavage site; for cleavage site 1 (S1/S2), reported residues 685 and 686 were defined as active residues for the S glycoprotein, whereas residues 815 and 816 were defined as active residues for cleavage site 2 (S′) [8]. Reported substrate binding residues 435, 460 and 462 [36] were defined as active residues of TMPRSS2 for docking. All docking simulations were performed using default parameters. For all docking simulations, only chain C (RBD is in ‘up’ position) was selected and used. The best model with the least Z- and HADDOCK scores and largest cluster size was selected for further assessment (Figure 1).

### 2.2. Mutagenesis, Binding Affinity and Interfacial Contact Predictions

A previous study has examined genetic susceptibility to COVID-19 by assessing DNA polymorphisms in *TMPRSS2* and *ACE* genes across different populations. The group identified 63 putative deleterious variants in the *TMPRSS2* coding region, in which the V160M variant is carried by all populations; interestingly, although D435Y, S460R and G462D/G462S have low allele frequencies, they are all carried only by the European population [21]. Nevertheless, D435, S460 and G462 are important residues involved in substrate binding. These unique variants in TMPRSS2 may potentially suggest explanations for differential susceptibility to SARS-CoV-2; thus, we selected all residues involved in the substrate binding for further analysis in this study. These residues were subjected to mutagenesis; they were mapped, modeled and analyzed in PyMOL using the crystal structure 7MEQ of human TMRPSS2, previously docked onto the S glycoprotein (6VSB).

Predicted binding energy (ΔG) and dissociation constant (*K*_D_) as well as interfacial contacts between the S/TMPRSS2 molecules were calculated using the PRODIGY web server [37], available at https://wenmr.science.uu.nl/prodigy/ (accessed on 12 December 2021). Different types of interfacial contacts such as charged–charged, charged–polar, charged–apolar, polar–polar, polar–apolar and apolar–apolar were identified. The wild-type (WT) and polymorphic variants of TMPRSS2 were analyzed, and their ΔG, *K*_D_ and surface contacts were determined.

### 2.3. TMPRSS2 and Protease Inhibitors Interaction Analysis

Previous studies have identified several potential protease inhibitors for COVID-19 treatment [8,31,38,39]. Camostat mesylate and nafamostat are a few protease inhibitors that have been suggested to inhibit the TMPRSS2 serine protease. In order to understand the mechanism of inhibition exhibited by these serine protease inhibitors, detailed intermolecular interactions between the protein inhibitors and TMPRSS2 are crucial. Thus, using the structures of camostat mesylate and nafamostat, retrieved from PubChem and converted into PDB files using PyMOL, protein-ligand docking analysis was performed using AutoDock Vina, as implemented in UCSF Chimera [40]. The crystal structure of TMPRSS2 (PDB: 7MEQ) was used as the receptor, whereas camostat mesylate and nafamostat were used as the ligand in the docking simulations. Molecular docking simulations were performed at the reported substrate binding sites D435, S460 and G462 by adopting the docking grid size of 20 × 20 × 25 Å along three axes, covering all the essential residues centered at −8.55, −2.05, 16.07 Å region, to provide enough space for the ligand conformations. At least 10 conformations were generated, and the model with the least binding energy and RMSD was chosen for further analysis. All generated docked structures were subjected to mutagenesis and visualized using PyMOL.

## 3. Results

### 3.1. TMPRSS2 Bound in Close Proximity to Two Cleavage Sites of the S Glycoprotein

**Figure 2:**
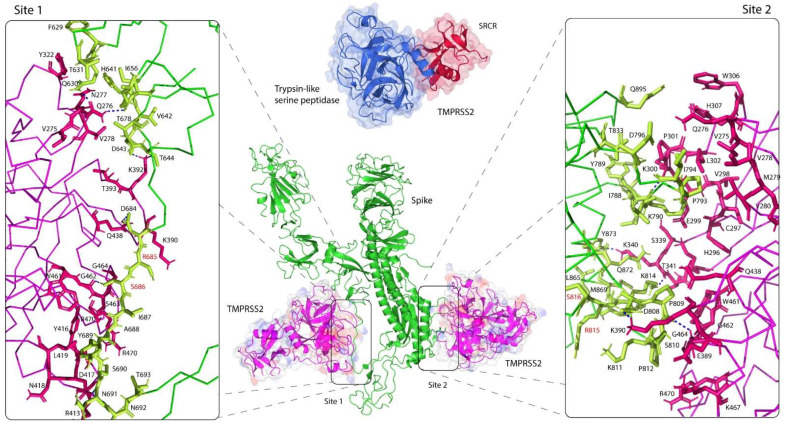
Molecular docking of TMPRSS2 with the spike glycoprotein. TMPRSS2 (PDB: 7MEQ) [31] bound in close proximity to cleavage sites 1 (R685/R686) (**left**) and 2 (R815/S816) (**right**) of the SARS-CoV-2 S glycoprotein (PDB: 6VSB) [33]. TMPRSS2 and the S protein were colored in magenta and green, respectively. Interactions between the two molecules were strengthened by formation of several hydrogen bonds; site 1: between Q276 of TMPRSS2 and Q630/G655 of the S protein, between K392 and D643/T644, between D417 and N691/N692, between Q438 and D684, and between G462 and S686; site 2: between K300 of TMPRSS2 and K790 of the S protein, between K340 and K814/Y873, between K390 and D808, and between G464 and P809. The crystal structure of TMPRSS2 (PDB: 7MEQ) [31], comprised of two subdomains, a class A scavenger receptor cysteine-rich (SRCR) domain (red) and a trypsin-like serine peptidase (blue).

**Figure 3:**
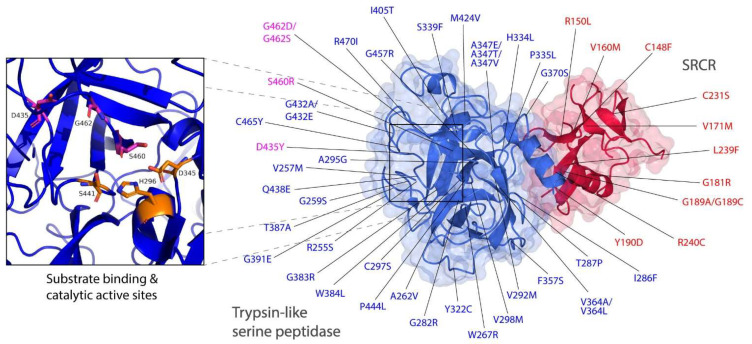
TMPRSS polymorphisms mapped on to the structure of human TMPRSS2 and its active sites. TMPRSS2 residues showing natural polymorphic variations across human populations [21] were mapped on to the structure of TMPRSS2 (PDB: 7MEQ) [31]. The SRCR domain was colored in red and the trypsin-like serine peptidase domain was colored in blue. Substrate binding (D435, S460 and G462) and catalytic active (H296, D345 and S441) sites were colored in magenta and orange, respectively.

TMPRSS2 is a membrane-anchored serine protease consisting of three main domains: an intracellular domain, a transmembrane domain and an active ectodomain. The ectodomain is comprised of three subdomains: a low-density lipoprotein receptor type A (LDLR-A) domain, a class A scavenger receptor cysteine-rich (SRCR) domain and a trypsin-like serine peptidase. The crystal structure of the recombinant human TMPRSS2 was recently published [31]. The 1.95-Å structure shows a biologically active ectodomain, in which the LDLR-A domain is absent (Figure 2). The serine peptidase domain is shown to adapt the common trypsin fold with two six-stranded β-barrels, converging to a central active site cleft, in which the triad of catalytic active residues H296, D345 and S441 and substrate binding sites D435, S460 and G462 are found sandwiched between the two folds (Figure 3).

To demonstrate the intermolecular interaction between the human TMPRSS2 and S proteins, we established *in silico* binding analysis and molecular docking between the two proteins. Two distinct docking simulations were performed for each cleavage site of the viral S protein, and TMPRSS2 was found to bind in close proximity to each site (Figure 2). The structures revealed that both cleavage sites were within the flexible loops of the S protein that interacted with both β-barrels of the serine peptidase domain of TMPRSS2 and were placed within the central active site cleft. The binding between the two molecules was strengthened by formation of several hydrogen bonds, particularly an 1.8 Å-hydrogen bond, formed between G462 of TMPRSS2 and S686 of the cleavage site one of the S protein. Although both cleavage sites were not in direct contact with the catalytic active sites of TMPRSS2, the functionally important residues of TMPRSS2 that formed the central cleft interacted with the residues of the S protein that immediately flanked the cleavage sites. These flexible loops may undergo conformational changes upon binding, which may bring both cleavage sites R685/S686 and R815/S816 closer to the catalytic active sites of TMPRSS2. This may subsequently activate proteolytic cleavage of S1/S2 and S’, and ultimately leading to host membrane fusion [8].

### 3.2. Intermolecular Interactions between TMPRSS2 Polymorphic Variants and the S Glycoprotein

Structural evaluation of ACE2 polymorphism has been previously reported [30]. The study has broadly classified ACE2 polymorphic variants into two categories based on their predicted effect on ACE2/RBD binding, on whether it could potentially increase or decrease the binding affinity of ACE2 to the SARS-CoV-2 S glycoprotein [30]. The study describes the structural basis of interactions between the human ACE2 polymorphic variants and S protein, which provides valuable information on the SARS-CoV-2 susceptibility. In order to evaluate the effect of the human TMPRSS2 polymorphic variants on receptor binding by the SARS-CoV-2 S glycoprotein, we modelled and mapped the previously identified natural TMPRSS2 polymorphic variants [21] using the recently published crystal structure of TMPRSS2 (PDB: 7MEQ) [31]. There were 63 putative deleterious variants in the *TMPRRS2* coding region [21], across different populations, of which 51 variants were distributed in SRCR and trypsin-like serine peptidase domains (Figure 3). Uniquely, distribution of deleterious variants in TMPRSS2 differed among nine populations: African and European populations carried 22 and 37 deleterious variants in TMPRSS2 respectively, whereas East and South Asian populations only carried four deleterious variants. Particularly, all nine populations carried V160M variants with high allele frequency. Interestingly, although D435, S460 and G462 residues are important for the substrate binding, D435Y, S460R and G462D/G462S variants were all carried by European population only [21].

**Figure 4:**
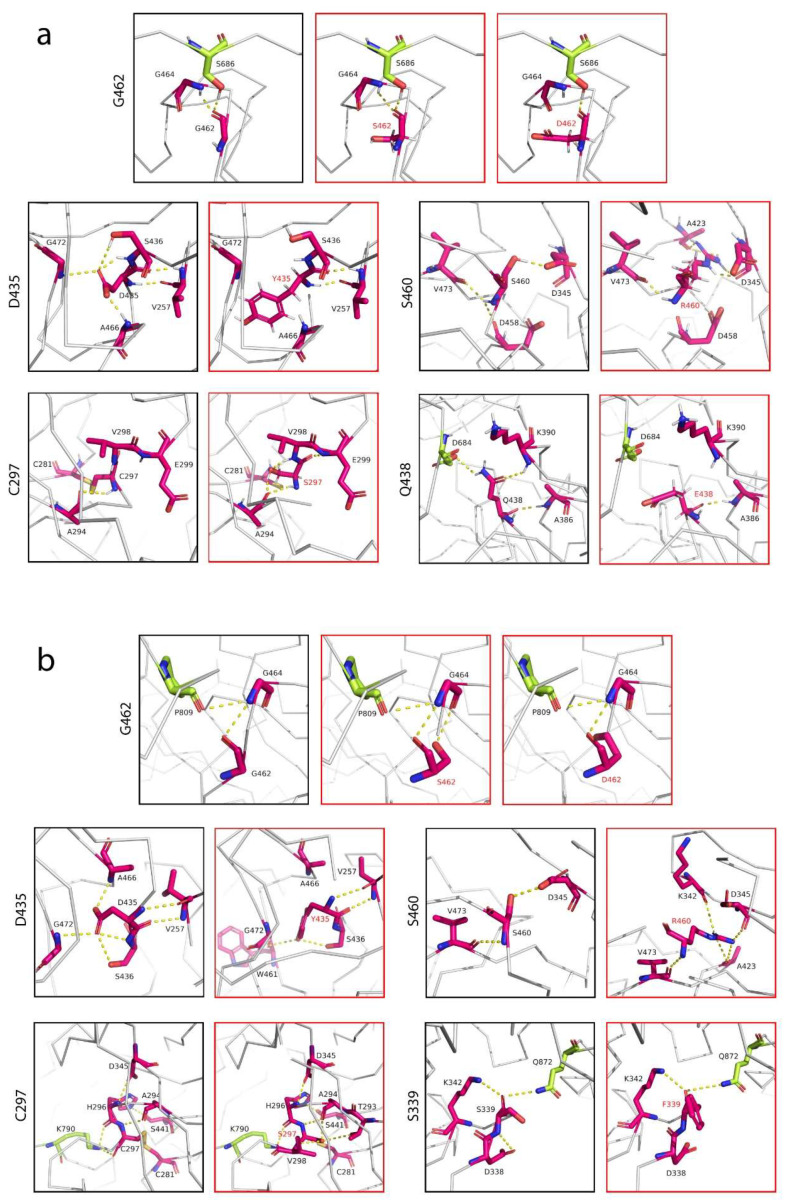
Interfacial contacts between the TMPRSS2 polymorphic variants and SARS-CoV-2 S glycoprotein. (**a**) Interactions at cleavage site 1 by residues G462, D435, S460, C297 and Q438. G462 made a direct contact with cleavage site 1 (residue S686), whereas Q438 established a contact with the residue D684 of the SARS-CoV-2 S glycoprotein, located a few residues away from the cleavage site. (**b**) Interactions at cleavage site 2 by residues G462, D435, S460, C297 and S339. Residues G462, C297 and S339 of TMPRSS2 made contacts with residues P809, K790 and Q872 of the S protein, respectively. TMPRSS2 residues were colored in magenta and the S protein residues were colored in lime green. Interaction mapping was performed using the structures of TMPRSS2 (PDB: 7MEQ) [31] and SARS-CoV-2 S glycoprotein (PDB: 6VSB) [33].

Given the importance of D435, S460 and G462 residues as substrate binding sites, we sought to evaluate the effect of TMPRSS2 polymorphisms on receptor binding by the S glycoprotein at cleavage sites one and two. Mutagenesis was performed for each residue involved in the receptor binding (Figure 3). Unlike ACE2, where most interactions that ACE2 makes with the RBD interface are mainly centered on its two α-helices [41], we found that TMPRSS2 interactions with the S protein were mostly centered on its extensive loop interface, which made contacts with the loop regions of the S protein (Figure 2). This feature would allow both the TMPRSS2 and S loop contact regions to extend and conform further towards each other, establishing more extensive contacts between the two interfaces. Thus, natural polymorphic variants of TMPRSS2 in this loop region could be utilized by the S protein loop, changing susceptibility to the viral recognition. By far the most important residue that made contacts with the cleavage site 1 was G462, as it is a part of the substrate binding triad. Based on its structure, G462 established polar contacts with its adjacent residue G464 and particularly S686 at the cleavage site 1 (Figure 4a). We predicted that either G462S or G462D would alter polar contacts with S686, leading to a change in the binding affinity between the SARS-CoV-2 and TMPRSS2. The introduction of a negatively-charged aspartate replacing glycine not only introduced a negative charge at position 462 but was also predicted to break the interaction with G464, whereas the introduction of a serine was predicted to have longer and weaker polar contacts. Although D435 was predicted to have no contacts with the S protein, D435 established an extensive network of polar contacts between its adjacent residues, all within the central active site cleft: S436, V257, G466 and G472. However, Y435 was then primed to disrupt these interactions, except with V257, where backbone interactions were still intact (Figure 4a). The predicted effect of the D435Y variant was increased hydrophobicity at the protein interface by replacing the negatively-charged aspartate with a hydrophobic residue, which could contribute to a change in binding affinity. The effect of natural S460R polymorphism, which replaced serine with the longer side-chained arginine had introduced a positive charge and therefore might contribute to an optimal interaction. C297 is an important residue as, together with C281, it formed a disulphide bond that stabilized the protein contact surface around the catalytic active sites. The natural C297S variant, however, which completely abolished this bond by introducing a serine, established new several polar contacts with its adjacent residues. Similar size to cysteine, replacement with a serine was small enough to accommodate a tight space between the residues and did not disrupt the formation of the loops. Although the disulphide bond was abolished, the variant introduced three additional contacts between the neighboring residues. Based on our predicted structure, apart from G462 that had direct contact with the S glycoprotein, Q438 of TMPRSS2 also established a polar contact with D684 of the S protein and interestingly, D684 was located next to cleavage site one. Q438 is one of the functionally important residues that interacts with D684, which then may bring cleavage site one closer to the catalytic active sites of TMPRSS2. Introduction of a glutamate replacing glutamine not only introduced a negative charge at position 438 but was also predicted to break the interaction with D684. The side chain of E438 is now primed away facing opposite of D684 due to the similarity of their charges.

TMPRSS2 interactions with the SARS-CoV-2 S glycoprotein at cleavage site two showed similar patterns to the interactions at cleavage site one, as discussed above. However, the substrate binding residue G462 did not make direct contact with the loop of the S protein; instead, its neighboring residue G464 served as its intermediary contact. Unlike interactions observed at cleavage site one, where G462 made a direct contact with S686, G462 established a polar contact with G464, which then made a direct contact with P809, located few residues away from cleavage site two (Figure 4b). This is possibly due to the fact that cleavage site two (R815/R816) is located at the junction between the loop and α-helix of the S protein, inaccessible without conformational changes, which may bring the cleavage site closer to the catalytic active sites of TMPRSS2. Similar to the interactions at cleavage site one above, we predicted that the natural G462D and G462S variants would change the binding affinity to the S protein in similar fashions discussed above. The effects of D435Y and S460R variants, similar to the interactions at cleavage site one, were increased hydrophobicity at position 435 and the introduction of a positive charge at position 460, respectively. C297 formed a series of hydrogen bonds with its neighbors and K790 of the S protein, as well as a disulphide bond with C281 (Figure 4b). The C297S variant, however, had completely abolished the disulphide bond but retained the contacts with the S protein, and the bonds were longer. We predicted this may well decrease the affinity between the two molecules. Another variant that established direct contact with the S protein was S339F, where it formed a hydrogen bond with Q872. Introduction of a phenylalanine replacing serine not only introduced a hydrophobic residue but was also predicted to have a stronger bond and therefore, would increase the binding affinity.

### 3.3. Altered Affinity of Natural TMPRSS2 Polymorphic Variants for the S Glycoprotein

In order to validate our structural predictions, we calculated the effect of selected natural TMPRSS2 polymorphic variants on its binding affinity for the SARS-CoV-2 S protein. Previously docked S protein and TMPRSS2 structures were subjected to mutagenesis and then used in the binding affinity (ΔG) and dissociation constant (*K*_D_) predictions using the PRODIGY web server [37]. We found that TMPRSS2 was predicted to bind to the SARS-CoV-2 S glycoprotein at cleavage site one (*K*_D_, 450 nM and ∆G, −8.6 kcal mol^−1^) (Table 1) less strongly compared to cleavage site two (*K*_D_, 27 nM and ∆G, −10.3 kcal mol^−1^) (Table 2), indicating a more extensive network of interacting loop regions at the cleavage site two compared to cleavage site one (Figure 2). This is consistent with the previous study that reported an increased TMPRSS2 affinity for cleavage site two, compared to cleavage site one [36]. We also measured the predicted affinity of the S glycoprotein for the natural TMPRSS2 polymorphic variants, described above. While the C297S TMPRSS2 variant had similar affinity to the WT TMPRSS2 at cleavage site one (*K*_D_, 450 nM vs. 450 nM), the C297S variant showed marginally lower predicted affinity for the S protein at cleavage site two (*K*_D_, 32 nM vs. 27 nM). The D435Y and S460R variants, however, showed similar predicted affinity to the WT TMPRSS2, either at cleavage sites one or two. We found that both the G462D and G462S variants had an apparent decrease in predicted binding affinity for the S protein at both cleavage sites. The G462D variant had significantly decreased affinity compared to the WT TMPRSS2 at cleavage sites one (*K*_D_, 680 nM vs. 450 nM) and two (*K*_D_, 33 nM vs. 27 nM). Similarly, the G462S variant had a decreased affinity at both sites (*K*_D_, 620 nM and 36 nM at cleavage sites one and two, respectively). Interestingly, while majority of the selected natural TMPRSS2 polmorphic variants showed a significant decrease in the predicted binding affinity, the Q438E and S339F variants, in contrast, showed higher affinities for the SARS-CoV-2 S glycoprotein. The Q438E variant showed an apparent increase in predicted binding affinity compared to the WT TMPRSS2 at cleavage site one (*K*_D_, 400 nM vs. 450 nM) (Table 1), whereas the S339F variant had a marginal increase affinity at cleavage site two (*K*_D_, 20 nM vs. 27 nM) (Table 2).

### 3.4. Selective Binding of Serine Protease Inhibitors to TMPRSS2

**Figure 5:**
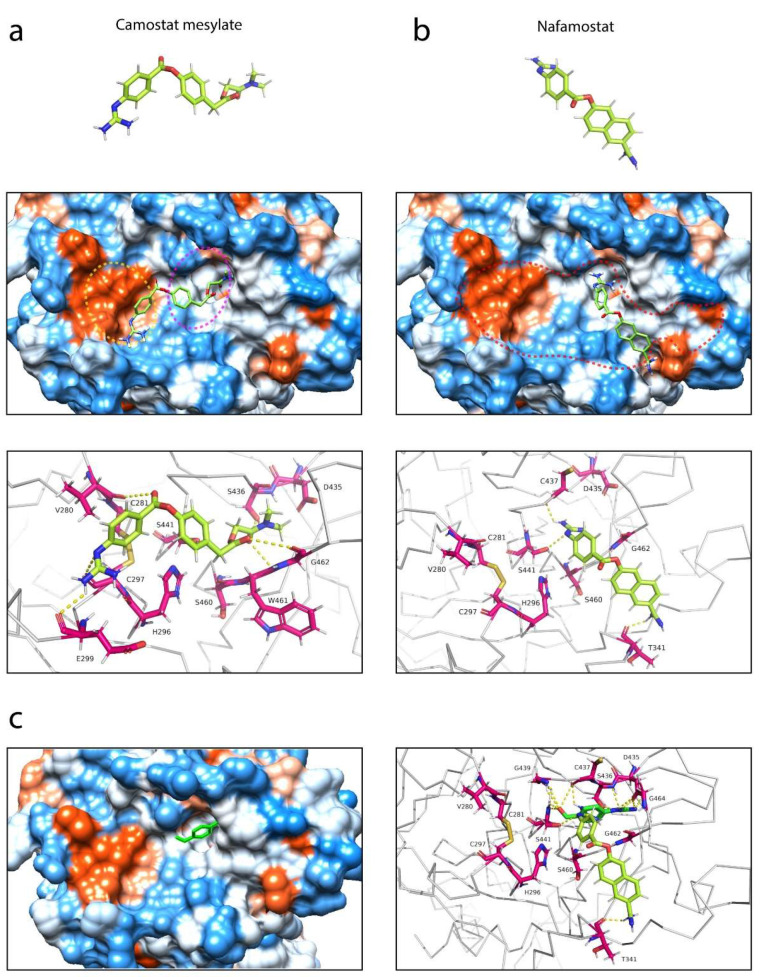
Structural basis of interactions between TMPRSS2 and two potential protease inhibitors. (**a**) Interactions of camostat mesylate with TMPRSS2. Camostat mesylate is a guanidino-containing, nonspecific serine protease inhibitor (top) and was positioned along the central active site cleft (middle) to shield both the substrate binding (circle, magenta) and catalytic active (circle, yellow) sites from access by the substrate, such as the SARS-CoV-2 S glycoprotein. Camostat mesylate established six polar contacts with the residues C281, C297, V280, S436, W461 and G462 of TMPRSS2 (bottom). (**b**) Interactions of nafamostat with TMPRSS2. Nafamostat is a guanidino- and amidino-containing, nonspecific serine protease inhibitor (top) and was positioned along the central cleft (circle, red) but at a different site, partially away from the catalytic active site (middle). Although less contacts formed, nafamostat established a hydrogen bond with S441, one of the catalytic active sites in addition of two strong hydrogen bonds with T341 and C437 (bottom). (**c**) Interactions of 4-guanidinobenzoic acid, a phenylguanidino moiety of nafamostat with TMPRSS2 (PDB: 7MEQ). Nafamostat rapidly acylates TMPRSS2 and slowly hydrolyzes, forming reversible phenylguanidino covalent complex with the catalytic serine residue S441 of TMPRSS2 [31]. Location of 4-guanidinobenzoic acid in the TMPRSS2 substrate binding pocket (left) and comparison of interactions between 4-guanidinobenzoic acid and nafamostat, colored in green and lime, respectively (right).

Several previous studies have reported potential serine protease inhibitors of TMPRSS2 for COVID-19 treatment [8,31,38,39]. Camostat mesylate and nafamostat are a few examples of protease inhibitors that have been suggested to inhibit TMPRSS2. In order to understand the mechanism of inhibition exhibited by these serine protease inhibitors, detailed intermolecular interactions between the protein inhibitors and TMPRSS2 are crucial. The structures of camostat mesylate and nafamostat were used in the protein-ligand analysis, performed using AutoDock Vina, as implemented in Chimera [40]. From the molecular docking simulations, we found that both inhibitors can fit into the central active site cleft of TMPRSS2, although in different orientations (Figure 5). The docked complex of TMPRSS2 with camostat mesylate showed that the protease inhibitor bound to the central cleft via hydrogen bonding interactions with the residues C281, C297, V280, S436, W461 and G462 (Figure 5a). Camostat mesylate was positioned along the cleft to shield both the substrate binding and catalytic active sites from access by the substrate. While camostat mesylate formed a strong polar contact with one of the residues present in the substrate binding triad, it did not establish contacts with any residues present in the catalytic triad such as H296, D345 and S441. However, it made a hydrogen bond with the residue C297, located next to H296. In contrast, the structure of TMPRSS2 in complex with nafamostat showed that the protease inhibitor bound to the central cleft at a different orientation, partially away from the catalytic active site (Figure 5b). However, unlike camostat mesylate—which was predicted to completely shield the catalytic site but did not interact with any of the catalytic residues—nafamostat had direct contact with S441 via its guanidino group. Similarly, nafamostat has been previously shown to slowly hydrolyze, forming reversible phenylguanidino covalent complex with S441 of TMPRSS2 (Figure 5c) [31]. Compared to camostat mesylate, which formed six hydrogen bonds, nafamostat only established three hydrogen bonds with TMPRSS2. The other two were with T341 and C437, and these had established strong hydrogen bonding interactions, predicted at 2.2 and 2.3 Å, respectively.

**Figure 6:**
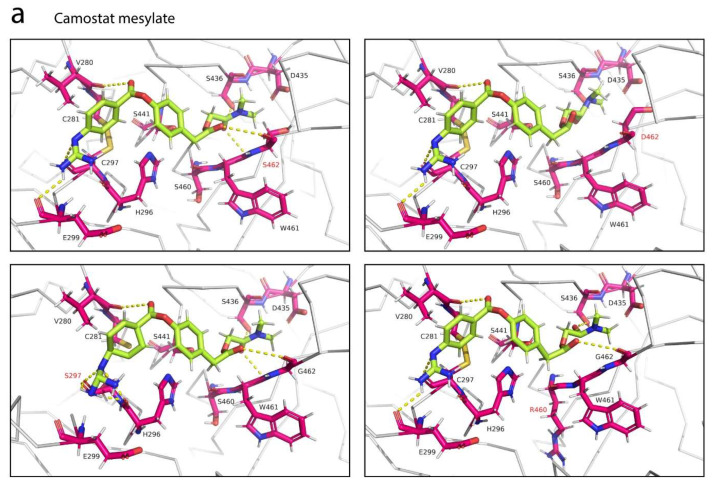

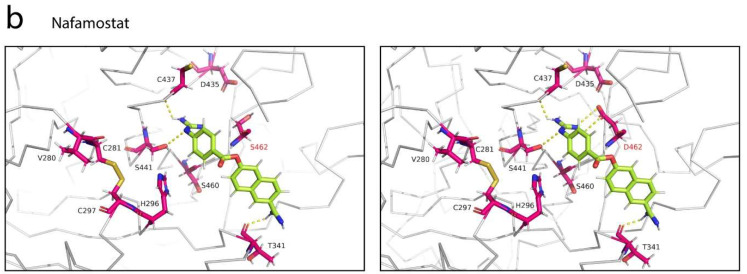
Analysis of contacts between TMPRSS2 polymorphic variants and two protease inhibitors. (**a**) Interactions of camostat mesylate with the natural TMPRSS2 polymorphic variants G462S, G462D, C297S and S460R. The G462S variant had little effect to the interactions between TMPRSS2 and camostat mesylate (top, left). Whereas, the G462D variant had completely abolished two polar contacts between TMPRSS2 and camostat mesylate (top, right). Introduction of a serine replacing cysteine at position 297 had abolished the contact between E299 of TMPRSS2 and the guanidino group of camostat mesylate, and had introduced additional four polar contacts (bottom, left). Introduction of an arginine at position 460 had abolished the contact between W461 and camostat mesylate (bottom, right). (**b**) Interactions of nafamostat with the natural TMPRSS2 polymorphic variants G462S and G462D. The G462S variant had no effect to the interactions between TMPRSS2 and nafamostat (left), whereas the G462D variant had established two hydrogen bonds between the guanidino group of nafamostat and D462 (right).

Given the importance of the natural TMPRSS2 polymorphic variants in the alteration of binding affinity for the S glycoprotein, we sought to evaluate the effect of these variations on interactions between TMPRSS2 and its protease inhibitors. Both docked structures of TMPRSS2 in complex with camostat mesylate and nafamostat were subjected to mutagenesis, and we found that the G462S variant did not alter the interactions between TMPRSS2 and both protease inhibitors (Figure 6). Introduction of a serine replacing glycine had insignificant effect to these interactions, probably due to the small and uncharged serine. The G462S variant had maintained its hydrogen bond with camostat mesylate (Figure 6a) and no interaction with nafamostat (Figure 6b) was observed. However, the introduction of an aspartate had completely abolished two hydrogen bonds between TMPRSS2 and camostat mesylate, which was mediated by W461 and G462. Unlike serine, aspartate is negatively-charged and has longer side chain that could repel away the dimethylamino group of camostat mesylate (Figure 6a). Interestingly, due to the different orientation of nafamostat binding to TMPRSS2, the G462D variant had established two additional polar contacts between the guanidino group of nafamostat and D462 (Figure 6b). While the introduction of a serine at position 462 had little effect on the interactions between TMPRSS2 and camostat mesylate, the introduction of the same residue at position 297, replacing cysteine, abolished the polar contact between the guanidino group of camostat mesylate and E299. Interestingly, the variant also introduced four additional polar contacts to the guanidino group—two hydrogen bonds from H296 and two hydrogen bonds from S297. The S460R variant, on the other hand, did not interact with camostat mesylate but instead caused disruption to the polar contact between W461 and the oxygen atom of camostat mesylate (Figure 6a).

## 4. Discussion

Elucidating the genetic components that determine COVID-19 susceptibility and severity would allow us to assess individuals for the stratification according to risk so that safe and effective therapeutics can be developed and prioritized for those at high risk [18]. Our enhanced understanding of the biological mechanisms at the molecular level becomes pivotal to guide us in the development of effective personalized therapeutics against the SARS-CoV-2 infection. The mammalian host genes have been continuously shaped by ongoing challenges imposed by viruses that lead to the host-virus evolutionary arms race, which could alter the host and viral proteins allowing both to enhance their fitness [42]. In this particular context, various studies have analyzed and identified human genetic factors that are associated with susceptibility to the SARS-CoV-2 infection and thus COVID-19 disease severity [18,21,30,43]. For instance, several studies have focused on *ACE2* polymorphisms that interact with the SARS-CoV-2 S glycoprotein and identified key changes in human genetics that rendered individuals susceptible to the SARS-CoV-2 infection [21,30,44]. Given the vital role of TMPRSS2 in the SARS-CoV-2 cell entry, *TMPRSS2* polymorphisms may influence individuals’ susceptibility to SARS-CoV-2. A previous study has examined DNA polymorphisms in the *TMPRSS2* gene across different populations. The group identified 63 putative deleterious variants in the *TMPRSS2* coding region, in which V160M variant (rs12329760) is carried by all populations with the highest allele frequency (~25%). Interestingly, although D435Y, S460R and G462D/G462S have low allele frequencies, they are all carried only by the European population [21]. Furthermore, previous studies have reported that the common V160M variant, which has a deleterious effect on the TMPRSS2 protease, may decrease susceptibility to SARS-CoV-2 and protect young males and elderly women from severe COVID-19 infection [45,46]. These unique variants in TMPRSS2 may potentially suggest explanations for differential susceptibility to SARS-CoV-2. As the high-resolution 1.95-Å crystal structure of TMPRSS2 has been recently published [31], further functional observations are warranted and so far, the role of polymorphisms in the human TMPRSS2 in susceptibility to SARS-CoV-2 has not been structurally evaluated. Thus, in this study, we comprehensively examined the natural human TMPRSS2 polymorphic variants, as previously reported [21], and identified variants that will possibly either enhance or decrease susceptibility to the SARS-CoV-2 infection. Using the recently published protein structure of TMPRSS2 [31], we performed structural modelling to identify interactions between TMPRSS2 and the SARS-CoV-2 S glycoprotein and structurally expounded in detail the intermolecular interactions of the natural TMPRSS2 polymorphic variants.

Here, we identified two potential natural polymorphic variants of TMPRSS2 that will likely reduce an individual’s susceptibility to SARS-CoV-2 and a variant that will likely enhance susceptibility. Based on our binding affinity prediction, the G462D and G462S variants had decreased affinities to the S glycoprotein, compared to the WT TMPRSS2, both at cleavage sites one (Table 1) and two (Table 2). G462 is an important residue as it is a part of the substrate binding triad [36]. Our structural investigation suggested that the introduction of a negatively-charged aspartate replacing non-charged glycine not only introduced a negative charge at position 462 but was also predicted to completely abolish the interaction with G464, whereas the introduction of a serine was predicted to have longer and weaker polar contacts. In contrast, the Q438E and S339F variants improved binding affinities to the S glycoprotein at cleavage sites one and two, respectively. Although the introduction of a glutamate replacing glutamine had abolished the hydrogen bond with D684 of the S protein, the replacement also introduced a negative charge at position 438, which probably increased the electrostatic interaction between the two molecules at cleavage site one (Figure 4a). The replacement of serine by a phenylalanine at position 339 increased its hydrophobicity and reduced the length of the polar contact, established to Q872 of the S protein (Figure 4b), and therefore, predicted to increase the binding affinity at cleavage site two. Besides, based on our binding affinity predictions, the Q438E and S339F variants had *K*_D_ of 400 nM and 20 nM, respectively, compared to the WT TMPRSS2 that had been predicted to have *K*_D_ of 450 nM and 27 nM at cleavage sites one and two, respectively. Thus, the Q438E and S339F variants will likely render individuals harboring these polymorphisms more susceptible to SARS-CoV-2 infection. Although our computational analysis revealed several important TMPRSS2 variants that may enhance or decrease susceptibility to SARS-CoV-2, further binding affinity confirmations by using protein–protein interaction analyses such as surface plasmon resonance, bio-layer interferometry, microscale thermophoresis or isothermal titration calorimetry are warranted to confirm the current analysis.

The ongoing COVID-19 pandemic has claimed the lives of nearly 5.5 million people globally (WHO). Although several vaccines have been approved and widely administered, the number of fatal cases is still increasing daily. The disease continues to challenge health care systems worldwide. Emergence of new SARS-CoV-2 variants that have rendered currently deployed vaccines less effective is concerning as it would hinder us from achieving herd immunity [9]. While mild cases of COVID-19 require only standard care with no therapeutics involved, the treatment of more severe cases of COVID-19 requires the use of effective antivirals. Unfortunately, there are currently no approved targeted therapeutics to treat the SARS-CoV-2 infection, although several small molecules for the COVID-19 treatment are in development [39,47,48]. Ideally, many therapeutics under development aim to block or inhibit the life cycle of the SARS-CoV-2 virus in order to halt its spread within the host cells. Small molecules such as chloroquine, captopril, atorvastatin, doxycycline, camostat, nafamostat, ivermectin and some others are currently being tested in clinical trials and based on targeting the early events of SARS-CoV-2 life cycle [39]. For instance, chloroquine is a 4-aminoquinoline derivative and usually used in the treatment of malaria [49,50]. However, chloroquine has been shown to block SARS-CoV-2 infection at a low concentration in Vero cells by inhibiting the production of sialic acid, which is important for the host ACE2 glycosylation [51,52]. Doxycycline is a semi-synthetic broad-spectrum antibiotic and is currently in clinical trials for the COVID-19 treatment. Doxycycline is capable to chelate zinc metal and potentially may inhibit matrix metalloproteinases that play a key role in the SARS-CoV-2 infection [53]. In addition, doxycycline and other tetracyclines have been predicted to inhibit SARS-CoV-2 main and papain-like proteases [54,55]. Moreover, doxycycline has been shown to have a high in vitro antiviral activity against SARS-CoV-2 [56]. Camostat and nafamostat are guanidino-containing, nonspecific serine protease inhibitors, and have been shown to potentially inhibit TMPRSS2 [8,31,32,57]. Recently, camostat mesylate and nafamostat have been shown to reduce pulmonary infection by SARS-CoV-2 in mouse models, with nafamostat exhibiting greater potency [38]. Both protease inhibitors are currently being studied in several clinical trials for COVID-19 [39].

Here, we examined the intermolecular interactions between TMPRSS2 and its two potential inhibitors, camostat mesylate and nafamostat, and we found that both serine protease inhibitors bound within the central active site cleft of TMPRSS2 in different orientations (Figure 5). Unlike nafamostat, which was positioned away from the catalytic active site, camostat mesylate was positioned along the central cleft to shield both the substrate binding and catalytic active sites from access by TMPRSS2. Camostat mesylate has been previously shown to bind to TMPRSS2 at a stronger binding affinity than nafamostat [58], possibly due to the formation of six hydrogen bonds between camostat mesylate and TMPRSS2 (Figure 5a). Although camostat mesylate established a polar contact with one of the substrate binding triads, it did not form contacts with any catalytic active residues (Figure 5a). Interestingly, nafamostat formed a strong hydrogen bond with S441, a catalytic active site via its guanidino group (Figure 5b), which may explain its enhanced potency over camostat mesylate [31] and its higher inhibition constant (*K*_i_) [58]. Furthermore, we investigated the effect of different natural TMPRSS2 polymorphic variants on these interactions. The G462S variant did not alter the interactions between TMPRSS2 and both protease inhibitors (Figure 6). However, the introduction of an aspartate completely abolished two hydrogen bonds between TMPRSS2 and camostat mesylate, whereas it established two additional hydrogen bonds between the guanidino group of nafamostat and D462 of TMPRSS2. In addition, the C297S and S460R variants established additional three and one contacts, respectively, with camostat mesylate (Figure 6).

## 5. Conclusions

Understanding the natural TMPRSS2 polymorphisms not only provides information on the susceptibility to SARS-CoV-2 but can also be utilized to develop high-affinity and specialized TMPRSS2 inhibitors. We found out that two TMPRSS2 variants (G462D and G462S) were predicted to be protective variants; meanwhile, Q438E and S339F variants were predicted to increase susceptibility to the SARS-CoV-2 infection. We also found that G462D, C297S and S460R variants had possibly altered the interactions with the two potential protease inhibitors, camostat mesylate and nafamostat. Our current computational analysis examined the important natural TMPRSS2 variations and their interactions with the S glycoprotein and two potential serine protease inhibitors, which could be useful to develop high-affinity and personalized drugs for the treating of COVID-19 patients.

## Figures and Tables

**Table 1:** SARS-CoV-2 predicted binding affinity for TMPRSS2 polymorphic variants at the cleavage site 1.

Table 2 Variant	Binding Energy (∆G (kcal mol^−1^))	*K*_D_ (nM) at 25.0 °C	*K*_D_ (nM) at 37.0 °C
WT	−8.6	450	800
D435Y	−8.6	460	820
S460R	−8.6	460	810
G462D	−8.4	680	1200
G462S	−8.5	620	1100
C297S	−8.7	450	780
Q438E	−8.7	400	710

**Table 2:** SARS-CoV-2 predicted binding affinity for TMPRSS2 polymorphic variants at the cleavage site 2.

TMPRSS2 Variant	Binding Energy (∆G (kcal mol^−1^))	*K*_D_ (nM) at 25.0 °C	*K*_D_ (nM) at 37.0 °C
WT	−10.3	27	53
D435Y	−10.3	28	54
S460R	−10.3	27	53
G462D	−10.2	33	65
G462S	−10.1	36	70
C297S	−10.2	32	62
S339F	−10.5	20	40

## Data Availability

All data relevant to this review is included in the text and references.
